# A Pathophysiology-Oriented Imaging Phenotype Framework for Nonobstructive Coronary Artery Disease

**DOI:** 10.3390/jcdd13040171

**Published:** 2026-04-18

**Authors:** Hongqun Du, Wenyue Chen, Hao Tian, Hong Huang, Yong Wu, Jun Liu, Hongyan Qiao

**Affiliations:** 1 Department of Medical Imaging, Affiliated Hospital of Jiangnan University, No. 1000 Hefeng Road, Wuxi 214122, China; 9862019276@jiangnan.edu.cn (H.D.); 18714439404@163.com (W.C.); 13636838796@163.com (H.T.); coreenh@163.com (H.H.); jswuyong1989@outlook.com (Y.W.); 2 Department of Emergency, Affiliated Hospital of Jiangnan University, Wuxi 214122, China; liujun12345647@outlook.com

**Keywords:** nonobstructive coronary artery disease, coronary computed tomography angiography, CT-derived fractional flow reserve, plaque burden, perivascular fat attenuation index, imaging phenotype, risk stratification

## Abstract

Nonobstructive coronary artery disease (NOCAD) is increasingly recognized as a heterogeneous condition characterized by diverse pathophysiological mechanisms despite the absence of flow-limiting stenosis. We sought to establish a rule-based dominant imaging phenotype framework integrating functional, structural, and inflammatory dimensions derived from multiparametric coronary computed tomography angiography (CCTA). In this retrospective cohort of 485 patients with NOCAD, CT-derived fractional flow reserve (CT-FFR), quantitative plaque burden and high-risk plaque features, and perivascular fat attenuation index (FAI) were assessed. Using predefined percentile thresholds and hierarchical rules, patients were categorized into function-, structure-, inflammation-dominant, or low-risk phenotypes. During a median follow-up of 36 months, 56 patients (11.5%) experienced major adverse cardiovascular events (MACE). After multivariable adjustment, function dominance was associated with the highest risk (hazard ratio [HR] 4.054, 95% confidence interval [CI] 1.984–8.281; *p* < 0.001), followed by structure dominance (HR 3.129, 95% CI 1.410–6.944; *p* = 0.005), whereas isolated inflammation dominance did not show a statistically significant independent association with events, with wide confidence intervals indicating limited precision. These findings suggest a graded pattern of prognostic associations across functional and structural abnormalities in NOCAD and support a phenotype-oriented interpretation of CCTA metrics reflecting distinct biological axes of coronary pathology.

## 1. Introduction

Nonobstructive coronary artery disease (NOCAD) has traditionally been regarded as a relatively benign angiographic finding; however, accumulating evidence over the past decade has challenged this assumption [1,2,3]. Patients with angina and nonobstructive coronary arteries experience substantial symptom burden and remain at non-negligible risk for major adverse cardiovascular events (MACE), underscoring the limitations of stenosis-based assessment alone [4,5]. Contemporary clinical frameworks, including the broader concepts of angina with no obstructive coronary artery disease (ANOCA) and ischemia with no obstructive coronary artery disease (INOCA), have further emphasized that the absence of flow-limiting stenosis does not equate to the absence of clinically relevant coronary pathology that nonobstructive coronary syndromes encompass heterogeneous underlying mechanisms [6,7,8]. A key challenge in the management of NOCAD lies in its pronounced pathophysiological heterogeneity. Rather than representing a uniform disease entity, NOCAD encompasses a spectrum of mechanisms, including diffuse atherosclerosis without focal obstruction, adverse plaque remodeling, functional impairment of coronary blood flow, and inflammation-driven plaque activity [9,10]. These processes may occur independently or coexist within the same patient, leading to heterogeneous clinical trajectories that are poorly captured by luminal narrowing alone. As a result, conventional anatomic descriptors offer limited discriminatory power for risk stratification in this population. This heterogeneity suggests that NOCAD should be interpreted not merely as a stenosis-negative entity but as a spectrum of biologically distinct pathophysiological states.

Advances in coronary computed tomography angiography (CCTA) have enabled noninvasive, multiparametric characterization of coronary atherosclerosis beyond stenosis severity. CT-derived fractional flow reserve (CT-FFR) provides lesion- and vessel-level functional assessment by estimating the hemodynamic impact of coronary lesions, and has demonstrated prognostic value even in patients without obstructive disease [11]. Quantitative plaque analysis allows detailed evaluation of plaque burden and high-risk plaque features, which reflect structural disease extent and vulnerability and have been consistently associated with adverse outcomes [12]. In parallel, the perivascular fat attenuation index (FAI) has emerged as an imaging biomarker of local coronary inflammation, capturing inflammatory signaling between the vessel wall and surrounding adipose tissue, with growing evidence linking elevated FAI to future cardiovascular events [13,14].

While prior CCTA studies in NOCAD have demonstrated that combining functional, structural, and inflammatory markers improves prognostic discrimination, most investigations have adopted a model-centered perspective focused on incremental predictive performance [12,15,16]. Such approaches, although valuable, do not fully address a clinically relevant question: when multiple imaging abnormalities coexist—as is frequently observed in NOCAD—which pathological process predominates in driving risk at the individual patient level? Incremental gains in model performance do not necessarily translate into mechanistic insight, nor do they clarify how different dimensions of coronary pathology should be interpreted in concert. Addressing this gap requires a shift from additive prediction toward phenotype-oriented interpretation. A phenotype-based framework that operationally identifies the predominant imaging abnormality may provide a more interpretable way to summarize the biological heterogeneity of NOCAD and facilitate mechanism-aligned risk stratification [17,18]. Rather than treating CT-FFR, plaque characteristics, and inflammatory markers as interchangeable predictors, such an approach acknowledges that these imaging features represent distinct disease dimensions with potentially different prognostic associations.

Accordingly, in the present study, we performed a phenotype-oriented analysis in patients with NOCAD using a rule-based dominant imaging phenotype framework integrating functional (CT-FFR), structural (plaque burden and high-risk plaque features), and inflammatory (FAI) dimensions derived from CCTA. The aims were to (1) characterize the independence and internal features of these imaging dimensions; (2) compare clinical outcomes across dominant imaging phenotypes; and (3) evaluate the robustness of phenotype classification under alternative threshold definitions. Through this phenotype-oriented framework, we sought to provide a biologically interpretable model of coronary disease heterogeneity in NOCAD that aligns imaging findings with underlying pathophysiological dimensions.

## 2. Materials and Methods

### 2.1. Study Design and Population

**Figure 1:**
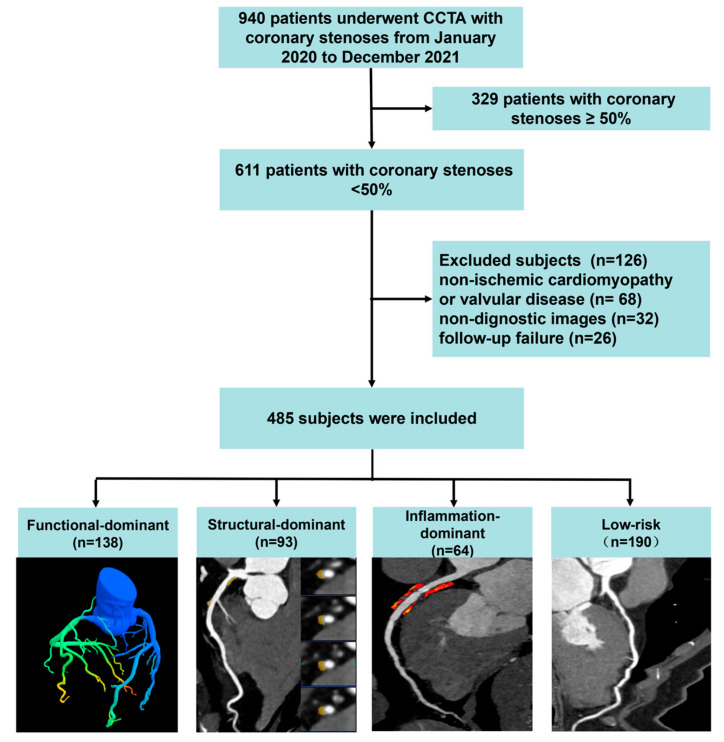
Study flowchart. Flowchart illustrating patient screening, inclusion and exclusion criteria, and final allocation into dominant imaging phenotype groups for outcome analysis.

This was a single-center, retrospective cohort study conducted in accordance with the Declaration of Helsinki and approved by the institutional ethics committee (approval number: LS2025211). The requirement for written informed consent was waived owing to the retrospective design and use of anonymized data. Consecutive patients who underwent clinically indicated CCTA between January 2020 and December 2021 were screened using the hospital information system and picture archiving and communication system. Patients were eligible for inclusion if CCTA demonstrated NOCAD, defined as the absence of ≥50% diameter stenosis in all major epicardial coronary arteries [19]. Exclusion criteria included: inadequate image quality due to severe motion artifacts; other types of cardiac diseases, including non-ischemic cardiomyopathy or valvular disease; and lost to clinical follow-up. The patient selection process is illustrated in Figure 1. Baseline demographic characteristics, cardiovascular risk factors, and medication use were extracted from electronic medical records. Standard clinical definitions were applied in accordance with contemporary guidelines.

### 2.2. CCTA Acquisition and Image Analysis

All CCTA examinations were performed using a dual-source CT scanner (Somatom Definition Flash, Siemens Healthineers, Erlangen, Germany) with electrocardiographic gating and standardized contrast injection protocols. Scan parameters included a tube voltage of 100–120 kVp, automatic tube current modulation, and a reconstructed slice thickness of 0.6 mm. Image acquisition and reconstruction were conducted in accordance with contemporary CCTA guidelines to ensure diagnostic image quality suitable for advanced quantitative analysis. Multiparametric CCTA analysis was performed using a commercially available artificial intelligence–based post-processing platform (Shukun Technology, Beijing, China), which enables integrated extraction of anatomical, structural, functional, and inflammatory imaging biomarkers from routine CCTA datasets. Following image reconstruction, CCTA datasets were imported into the AI platform for automated coronary artery segmentation and centerline extraction across the entire coronary tree. Quantitative plaque analysis was performed in an automated manner, yielding total plaque volume (PV) and vessel volume, from which plaque burden (PB) was calculated as the ratio of PV to vessel volume. High-risk plaque (HRP) was defined by the presence of at least two established adverse plaque features, including low-attenuation plaque, positive remodeling, napkin-ring sign, or spotty calcification [20]. CT-FFR was computed directly from routine CCTA images using an AI-assisted reduced-order computational framework. For each patient, the minimum CT-FFR value across the entire coronary tree was recorded as a global indicator of coronary functional impairment [21,22]. Perivascular inflammation was assessed using the perivascular FAI, automatically quantified as the mean CT attenuation of perivascular adipose tissue surrounding the major epicardial coronary arteries within predefined attenuation thresholds (−190 to −30 HU), reflecting local vascular inflammatory activity [23]. All AI-derived imaging parameters were reviewed for quality control by experienced cardiovascular radiologists who were blinded to clinical data and outcomes. Inter-observer agreement for CCTA-derived plaque characteristics, perivascular FAI, and CT-FFR measurements was assessed in a random sample of 50 patients by two independent readers (H.D. and W.C., respectively). Intra-observer reproducibility was evaluated in 30 cases by repeat measurement after an 8-week interval. Agreement was quantified using intraclass correlation coefficients (ICCs).

### 2.3. Data Preprocessing and Quality Control

Prior to analysis, data preprocessing and quality control procedures were applied to ensure the integrity and consistency of the combined clinical and imaging datasets. Patient identifiers, examination dates, follow-up duration, and event timing were cross-checked for internal consistency. Continuous variables were examined for plausibility and distributional characteristics. Variables with less than 5% missing values were handled using complete-case analysis. For variables with 5% or greater missingness, multiple imputation by chained equations was performed under the assumption of missing at random. Twenty imputed datasets were generated, and parameter estimates were pooled using Rubin’s rules to obtain final model coefficients and standard errors. Extreme values were assessed using boxplots and distributional histograms; when appropriate, winsorization was applied to reduce the influence of outliers while preserving the overall distribution. All preprocessing steps were completed prior to phenotype construction and outcome analysis.

### 2.4. Construction of Dominant Imaging Phenotypes

Imaging phenotypes were defined a priori without reference to clinical outcomes to minimize outcome-driven bias. In the absence of a widely accepted external cut-off system capable of jointly integrating CT-FFR, PB/HRP, and FAI into a unified phenotype framework, we adopted a prespecified, rule-based, and outcome-independent internal standardization strategy. Thresholds for CT-FFR, total PB, and perivascular FAI were determined using the 25th and 75th percentiles of their respective distributions within the study population. A hierarchical, rule-based framework was applied to classify each patient into a single dominant imaging phenotype: (1) Function-dominant phenotype: CT-FFR below the 25th percentile; (2) Structure-dominant phenotype: total plaque burden at or above the 75th percentile or the presence of HRP, in the absence of functional dominance; (3) Inflammation-dominant phenotype: perivascular FAI at or above the 75th percentile when neither functional nor structural criteria were met; (4) Low-risk phenotype: patients who did not meet any of the above criteria. This hierarchical classification ensured mutual exclusivity of phenotypes and was intended to improve interpretability. However, because functional, structural, and inflammatory abnormalities may coexist in the same patient, this rule-based assignment may simplify overlapping pathophysiological processes and introduce some degree of misclassification. Percentile-based thresholds were adopted as a prespecified and pragmatic approach for exploratory phenotype construction, allowing relative stratification of abnormalities within the cohort while avoiding post hoc threshold optimization. Because absolute values of CT-FFR, PB, and perivascular FAI may vary across imaging platforms, reconstruction methods, and study populations, internally derived percentile thresholds were considered more appropriate for this initial phenotype-oriented analysis than imposing a single universal cutoff across domains. To further examine the robustness of this approach, sensitivity analyses were performed using alternative percentile definitions (P67/P33 and P80/P20). These alternative percentile thresholds were used to evaluate the robustness of phenotype classification rather than to establish clinically applicable cut-off values.

### 2.5. Clinical Outcome Ascertainment

Clinical outcomes were ascertained through systematic review of electronic medical records, outpatient clinic documentation, and structured telephone interviews. Follow-up duration was calculated from the date of index CCTA examination to the occurrence of an endpoint or the last known clinical contact. The primary outcome was the occurrence of MACE, defined as a composite of all-cause death, nonfatal myocardial infarction, urgent coronary revascularization, or hospitalization for unstable angina. Each component event was adjudicated according to standardized definitions by investigators blinded to imaging phenotype classification [24]. When multiple events occurred in the same patient, only the first event was considered for time-to-event analyses.

### 2.6. Statistical Analysis

Baseline clinical characteristics and imaging parameters were summarized using descriptive statistics. Continuous variables are presented as mean ± standard deviation or median (interquartile range), depending on data distribution, while categorical variables are expressed as counts and percentages. Differences in imaging parameters across dominant imaging phenotypes were assessed using the Kruskal–Wallis test. Associations among CT-FFR, total PB, and perivascular FAI were evaluated using Spearman’s rank correlation coefficients. Time-to-event analyses were performed to evaluate the association between imaging phenotypes and clinical outcomes. Kaplan–Meier survival curves were generated to compare the cumulative incidence of MACE across imaging phenotypes, with between-group differences assessed using the log-rank test. Cox proportional hazards regression models were constructed to estimate hazard ratios (HRs) and 95% confidence intervals (CIs), with stepwise adjustment for clinically relevant covariates. Model assumptions were systematically evaluated. The proportional hazards assumption was tested using Schoenfeld residuals, and multicollinearity among covariates was assessed using variance inflation factors. Time-to-event analyses were performed using Cox proportional hazards regression. The dominant imaging phenotype was modeled as a four-level categorical variable, with the low-risk phenotype as the reference category, corresponding to three regression parameters. Given the limited number of outcome events, multivariable adjustment was intentionally restricted to a parsimonious set of five prespecified clinically relevant covariates (age, sex, hypertension, diabetes mellitus, and baseline statin therapy) to reduce the risk of overfitting. Accordingly, Model 1 included phenotype plus age and sex (5 regression parameters in total), Model 2 additionally included hypertension and diabetes mellitus (7 parameters), and Model 3 further included baseline statin therapy (8 parameters). Events-per-variable (EPV) ratios were formally assessed to evaluate model complexity in relation to the number of events. As a sensitivity analysis, Firth penalized Cox regression was performed for the unadjusted and sequentially adjusted models used in the primary analysis to reduce potential small-sample bias related to the limited number of outcome events. Hazard ratios, 95% confidence intervals, and *p* values were estimated using the same phenotype coding and covariate structure as in the main Cox models. In addition, bootstrap resampling was used to examine the stability of key phenotype-related regression coefficients across repeated samples. Bootstrap internal validation (500 resamples) was performed to assess calibration optimism and coefficient stability. Sensitivity analyses were also conducted using alternative percentile-based thresholds to examine the robustness of imaging phenotype classification and its association with outcomes. All statistical tests were two-sided, and a *p*-value < 0.05 was considered statistically significant. Statistical analyses were performed using R version 4.3.0 (R Foundation for Statistical Computing, Vienna, Austria).

## 3. Results

### 3.1. Baseline Characteristics of the Study Population

A total of 485 patients were included in the final analysis, of whom 56 (11.5%) experienced MACE during follow-up, while 429 (88.5%) remained event-free. The mean age of the overall cohort was 68.2 ± 7.2 years, and 43.7% were male. Baseline demographic characteristics, traditional cardiovascular risk factors and medication use were comparable between patients with and without MACE (all *p* > 0.05; Table 1). Specifically, there were no significant differences in age, sex distribution, BMI, or the prevalence of hypertension, diabetes mellitus, hyperlipidemia, smoking history, or family history of coronary artery disease.

Inter- and intra-observer reproducibility for quantitative plaque characteristics, FAI, and CT-FFR was good to excellent, with ICC values ranging from 0.748 to 0.953. With respect to conventional anatomic CCTA parameters, the degree of luminal stenosis, lesion length, and minimum lumen area showed no statistically significant differences between the MACE and non-MACE groups. In contrast, several advanced CCTA-derived metrics demonstrated significant differences. Patients who experienced MACE exhibited higher total PV (256.9 mm3 vs. 208.6 mm3, *p* = 0.005), PB (53.1 vs. 48.2, *p* = 0.005), a greater prevalence of HRP (19.6% vs. 3.7%, *p* < 0.001), and significantly lower CT-FFR values (0.86 vs. 0.91, *p* < 0.001) compared with those without MACE (Table 2). These findings suggest that functional impairment and plaque characteristics, rather than stenosis severity alone, were associated with adverse outcomes in this cohort.

### 3.2. Construction and Distribution of Dominant Imaging Phenotypes

**Figure 2:**
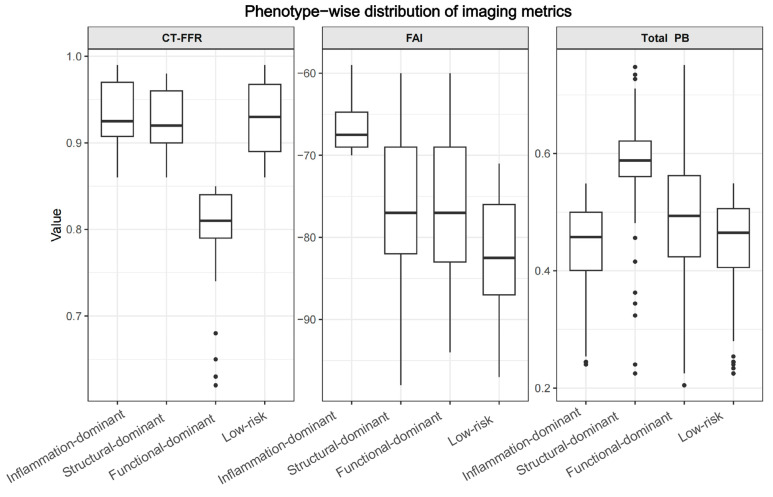
Distribution of imaging parameters across dominant phenotypes. Boxplots illustrate the distributions of CT-FFR, PB, and perivascular FAI among phenotype groups. The dots indicate outliers beyond the whiskers of the boxplots. Significant differences were observed across phenotypes for all parameters, demonstrating effective internal discrimination of the phenotype classification.

Using predefined percentile-based thresholds and a hierarchical classification strategy, patients were assigned to dominant imaging phenotypes at baseline without reference to follow-up outcomes. Among the 485 patients, 138 (28.5%) were classified as function-dominant, 93 (19.2%) as structure-dominant, 64 (13.2%) as inflammation-dominant, and 190 (39.2%) as low-risk. As illustrated in Figure 2, the distribution of imaging parameters across phenotypes aligned with the intended conceptual framework. CT-FFR values were lowest in the function-dominant group (0.810 [0.790, 0.840]), compared with 0.920 [0.897, 0.945] in the structure-dominant group, 0.925 [0.904, 0.958] in the inflammation-dominant group, and 0.930 [0.890, 0.962] in the low-risk group (Kruskal–Wallis *p* < 0.001). Structural abnormalities were most pronounced in the structure-dominant phenotype, which demonstrated the highest total PB (58.8 [56.0, 62.3]) and the greatest prevalence of HRP (17/93, 18.3%), compared with 7.2% in the function-dominant group. Perivascular FAI values were highest in the inflammation-dominant group (−67.5 [−69.0, −64.4] HU, compared with −77.0 [−83.1, −69.0] HU in the function-dominant group, −77.0 [−82.0, −69.0] HU in the structure-dominant group, and −82.5 [−87.0, −76.0] HU in the low-risk group (all *p* < 0.001). The low-risk group exhibited relatively preserved functional, structural, and inflammatory profiles. Differences in CT-FFR, total PB, and FAI among the four groups were all statistically significant. Spearman correlation analysis demonstrated weak and statistically non-significant associations among CT-FFR, plaque burden, and FAI (FAI vs. PB: r = 0.012, *p* = 0.79; FAI vs. CT-FFR: r = −0.031, *p* = 0.49; PB vs. CT-FFR: r = −0.055, *p* = 0.22).

Because functional, structural, and inflammatory abnormalities may coexist in patients with NOCAD, we further examined overlap across domains. Among the 485 patients, 190 (39.2%) had no abnormality, 194 (40.0%) had a single abnormality, 87 (17.9%) had abnormalities in two domains, and 14 (2.9%) had abnormalities in all three domains. Thus, overlap was present but involved only a minority of patients. To assess whether the mutually exclusive classification materially influenced the main findings, we performed a non-mutually exclusive sensitivity Cox analysis by entering functional, structural, and inflammatory abnormalities simultaneously as independent variables rather than redefining new phenotype groups. The functional and structural domains remained directionally consistent and statistically significant, supporting the relative stability of the main findings, whereas the inflammatory domain remained non-significant (Supplementary Figure S1).

### 3.3. Clinical Outcomes According to Dominant Imaging Phenotypes

**Figure 3:**
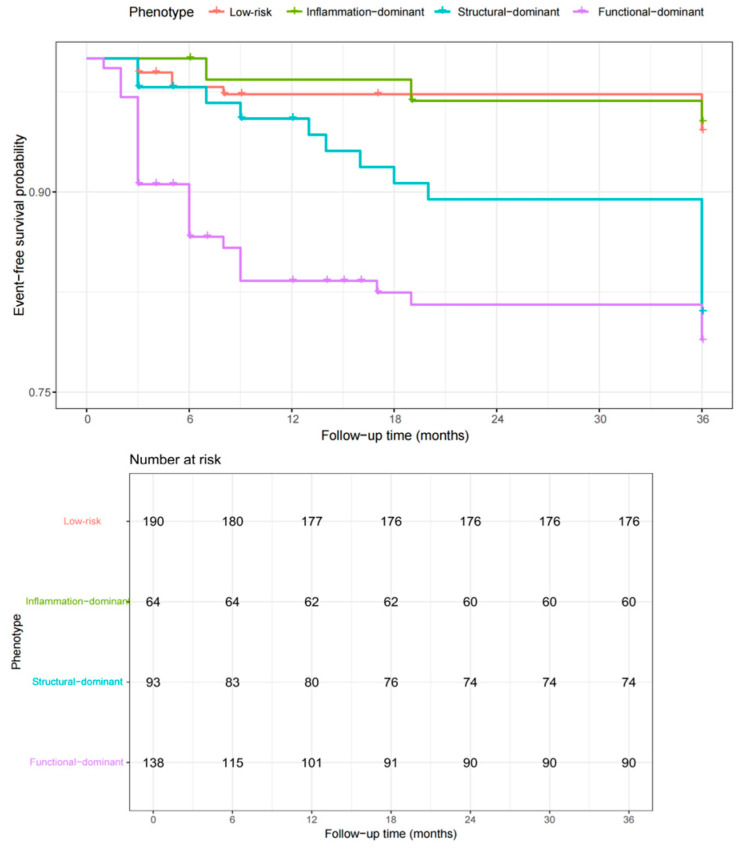
Kaplan–Meier curves for MACE by dominant imaging phenotype. Cumulative MACE-free survival differed significantly among phenotype groups, with the function-dominant phenotype demonstrating the highest event rate and the low-risk group showing the most favorable prognosis.

The median follow-up duration was 36 months. Kaplan–Meier analysis revealed significant differences in cumulative event-free survival among the dominant imaging phenotypes (log-rank *p* < 0.001; Figure 3). The function-dominant phenotype was associated with the highest event rate, followed by the structure-dominant phenotype, whereas the inflammation-dominant phenotype demonstrated event-free survival intermediate between the structure-dominant and low-risk groups on unadjusted analysis. Among the 485 patients, the low-risk group included 190 patients with 10 events (5.3%, 95% CI 2.6–9.5%), the inflammation-dominant group included 64 patients with 3 events (4.7%, 95% CI 1.0–13.1%), the structure-dominant group included 93 patients with 16 events (17.2%, 95% CI 10.2–26.4%), and the function-dominant group included 138 patients with 27 events (19.6%, 95% CI 13.3–27.2%). At 36 months, the estimated cumulative event rates were approximately 22% in the function-dominant group, 14% in the structure-dominant group, 11% in the inflammation-dominant group, and 7% in the low-risk group.

**Figure 4:**
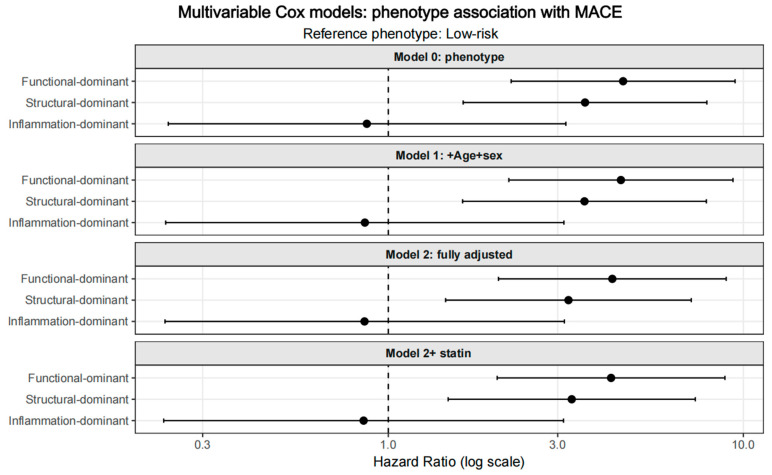
Multivariable Cox regression analysis of dominant phenotypes. HRs and 95% CI are shown for unadjusted and multivariable-adjusted Cox proportional hazards models. The low-risk phenotype served as the reference category. The models were sequentially adjusted for age and sex (Model 1), traditional cardiovascular risk factors including hypertension and diabetes (Model 2), and baseline statin therapy (Model 3).

In multivariable Cox regression analyses, a graded pattern of risk was observed across dominant imaging phenotypes (Figure 4). Using the low-risk group as the reference, the function-dominant phenotype was consistently associated with a significantly increased risk of MACE across all models. In the fully adjusted model, which included demographic variables and traditional risk factors, the function-dominant phenotype remained independently associated with MACE (HR = 4.054, 95% CI: 1.984–8.281, *p* < 0.001). The structure-dominant phenotype was also associated with a significantly elevated risk (HR = 3.129, 95% CI: 1.410–6.944, *p* = 0.005). In contrast, the inflammation-dominant phenotype did not reach statistical significance after adjustment (HR = 0.997, 95% CI: 0.314–3.162, *p* = 0.996). However, the wide confidence interval and the small number of events in this subgroup indicate limited precision, and this finding should therefore be interpreted cautiously rather than as evidence of no prognostic relevance. Importantly, additional adjustment for statin therapy did not materially alter the direction or relative magnitude of the observed associations, indicating that the risk stratification conveyed by dominant imaging phenotypes was independent of baseline statin use. Sensitivity analysis using Firth penalized Cox regression showed similar hazard ratio directions and comparable effect sizes, supporting the stability of the main findings despite the limited number of events (Supplementary Table S1). Sensitivity analyses using alternative percentile definitions yielded a generally consistent relative risk ordering across phenotype groups, supporting the internal robustness of the framework. However, these internally derived thresholds should be interpreted as part of a robustness analysis rather than as clinically validated decision cut-offs.

### 3.4. Sensitivity Analyses Using Alternative Threshold Definitions

**Figure 5:**
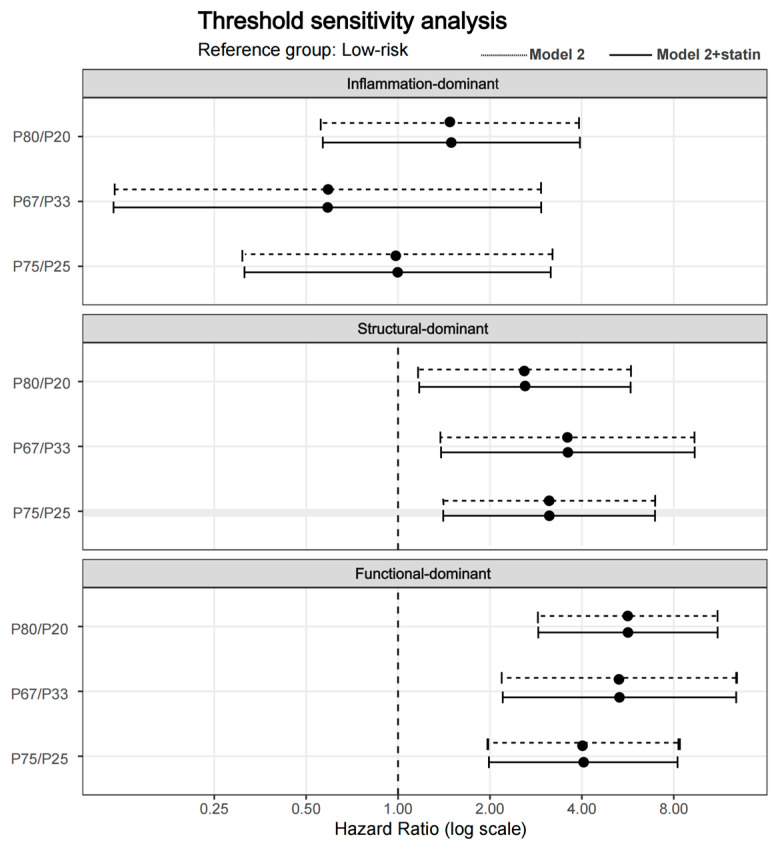
Sensitivity analysis using alternative percentile thresholds. Forest plots display hazard ratios for MACE across different threshold schemes (P75/P25, P67/P33, and P80/P20). The vertical dotted line represents the no-effect reference line at HR = 1.00.

**Figure 6:**
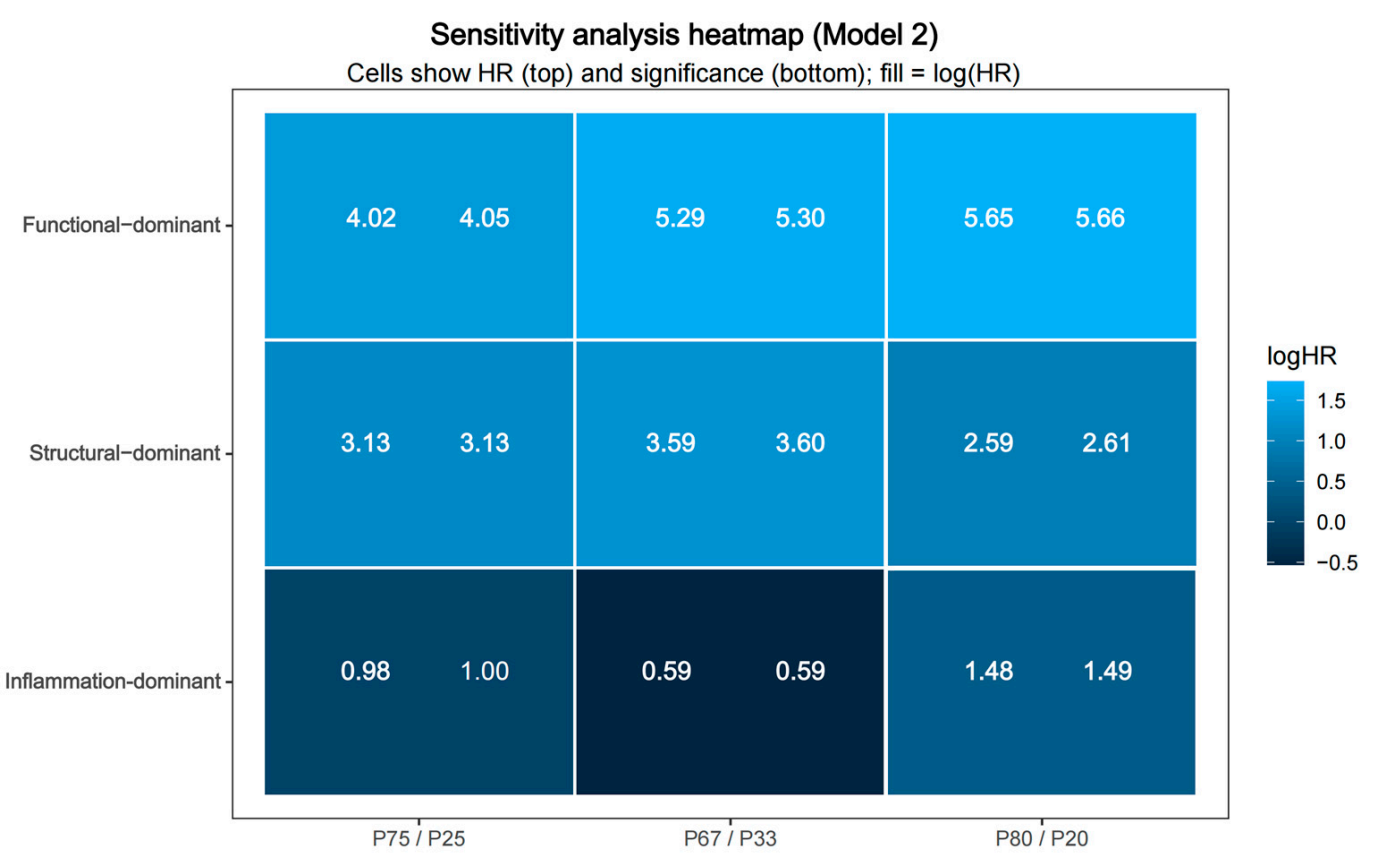
Heatmap of log-transformed hazard ratios across models and thresholds. Colors represent the magnitude and direction of log (HR) values derived from multivariable Cox models under different threshold definitions. Color intensity represents the magnitude and direction of association, with positive log (HR) values indicating increased risk relative to the low-risk reference group.

To assess the robustness of the dominant phenotype classification, sensitivity analyses were performed using alternative percentile thresholds (P67/P33 and P80/P20) for CT-FFR, total PB, and perivascular FAI. Across all threshold schemes, the relative risk ordering of the dominant phenotypes remained consistent (Figure 5). Under each alternative definition, the function-dominant phenotype consistently demonstrated the highest risk of MACE (HR range 4.02–5.66, all *p* < 0.001), followed by the structure-dominant phenotype (HR range 2.59–3.60, all *p* < 0.05). In contrast, the inflammation-dominant phenotype showed effect estimates fluctuating around unity with wide confidence intervals and no statistically significant associations across models (0.59–1.49, all *p* > 0.05). Importantly, no reversal of hazard ratio direction was observed for the function- or structure-dominant phenotypes across threshold definitions, further supporting the internal consistency of the framework. A heatmap of log-transformed hazard ratios further illustrated the stability of effect direction and relative magnitude across models and threshold definitions (Figure 6). The functional- and structure-dominant phenotypes consistently demonstrated positive log (HR) values across all cutoff schemes, supporting a consistent direction of association. In contrast, the inflammation-dominant phenotype showed effect estimates fluctuating around unity, without consistent directionality or statistical significance. Sensitivity analyses using alternative percentile definitions yielded a generally consistent relative risk ordering across phenotype groups, supporting the internal consistency of the classification approach.

## 4. Discussion

In this study, we developed a rule-based dominant imaging phenotype framework integrating functional, structural, and inflammatory metrics derived from CCTA in patients with NOCAD. The present study supports the concept that functional, structural, and inflammatory abnormalities represent partially independent biological axes in NOCAD. Within this cohort, the strongest prognostic association was observed for the function-dominant phenotype, followed by the structure-dominant phenotype, whereas the inflammation-dominant phenotype did not show an independent association after multivariable adjustment.

More broadly, the present framework may be interpreted within the evolving spectrum of nonobstructive coronary syndromes, including ANOCA and INOCA, which are increasingly recognized as heterogeneous conditions with mechanisms extending well beyond focal epicardial stenosis [25,26]. Within this broader perspective, the prognostic prominence of the function-dominant phenotype in our cohort is biologically plausible.

In nonobstructive coronary disease, reduced CT-FFR may reflect impaired coronary flow regulation and microvascular dysfunction, both relevant to ischemia and adverse outcomes in ANOCA/INOCA [27]. In the present cohort, patients classified as having a function-dominant phenotype, defined by reduced CT-FFR, consistently demonstrated the highest event risk, even after adjustment for demographic characteristics, traditional cardiovascular risk factors and baseline medication use. This finding is consistent with prior studies demonstrating the independent prognostic value of CT-FFR across a broad spectrum of coronary artery disease severity, including patients without flow-limiting stenosis [11,28,29]. CT-FFR reflects the hemodynamic consequences of coronary atherosclerosis by integrating lesion geometry, vessel morphology, and downstream microvascular resistance [30]. Unlike anatomic stenosis severity, which represents a static morphological descriptor, CT-FFR captures the physiological impact of lesions on coronary blood flow under simulated hyperemic conditions. Together, these findings suggest that reduced CT-FFR may represent the phenotype component most strongly associated with adverse outcomes in this framework.

The structure-dominant phenotype, characterized by elevated plaque burden and/or the presence of high-risk plaque features, was associated with a significantly increased risk of MACE, although the magnitude of risk was lower than that observed in the function-dominant group, consistent with prior studies suggesting that functional ischemia may be associated with a stronger short-term prognostic signal than structural plaque characteristics alone [31]. PB reflects the global extent of atherosclerotic involvement and vascular remodeling, while high-risk plaque features have been linked to plaque instability and future events in prior CCTA studies [32,33]. The comparatively lower risk associated with structural dominance may reflect the temporal nature of plaque-related risk. Structural abnormalities often represent a substrate for future instability and progression rather than an immediate trigger of ischemic events. In the absence of concomitant functional impairment, structurally high-risk plaques may contribute predominantly to intermediate- or long-term risk through gradual plaque evolution rather than acute flow limitation. These findings suggest that structural dominance may identify a subgroup of patients with a high atherosclerotic burden who are particularly amenable to intensified plaque-stabilizing strategies and longitudinal monitoring.

In contrast, the inflammation-dominant phenotype did not show a statistically significant independent association with short- to mid-term risk within the constraints of the present sample size. However, this finding should not be interpreted as evidence that coronary inflammation lacks biological or prognostic relevance. Rather, the wide confidence intervals indicate limited statistical precision, likely related to the relatively small size of the inflammation-dominant subgroup and the modest number of events. Accordingly, the present data are insufficient to exclude a clinically meaningful association, and longer follow-up or larger external cohorts may be required to better define the prognostic implications of inflammation-dominant phenotypes. Across alternative threshold definitions, effect estimates remained close to unity with wide confidence intervals, reflecting limited statistical precision and a relatively small number of events in this subgroup. Perivascular FAI has been proposed as a surrogate marker of local coronary inflammation, reflecting changes in adipocyte lipid content and tissue composition induced by inflammatory signaling from the vessel wall [34]. The modest prognostic signal observed in this study may indicate that inflammation-dominant findings represent an earlier or more transitional phase of disease rather than an immediate driver of short-term clinical events.

A key methodological observation of this study is the minimal correlation among CT-FFR, PB, and FAI, indicating that these metrics capture largely independent aspects of coronary pathology. This independence supports a multidimensional conceptualization of CAD, in which hemodynamic dysfunction, plaque accumulation, and inflammatory activation represent distinct yet interacting biological processes. Unlike incremental modeling approaches, such as radiomics-enhanced CCTA analyses that focus on improving discriminatory performance, the present framework emphasizes biological interpretability and dominant pathological axes [35]. By explicitly assigning dominance based on hierarchical rules, the proposed phenotype framework prioritizes interpretability and limits model complexity compared with more flexible data-driven approaches. The consistency of the observed association pattern across multiple threshold definitions further supports the internal robustness of this rule-based strategy.

Our findings are consistent with recent evidence suggesting that structural plaque characteristics and physiological impairment provide complementary prognostic information in coronary artery disease [36]. In particular, a recent systematic review and meta-analysis demonstrated that high-risk plaque features and impaired coronary physiology in non-culprit lesions provide synergistic prognostic information, supporting the concept that structural and functional abnormalities represent interacting but nonredundant domains of coronary risk [37]. From a clinical perspective, the proposed framework may serve as an adjunctive tool for integrated risk stratification rather than as a direct treatment algorithm. By identifying the dominant abnormal domain, it may support a more mechanism-oriented interpretation of multiparametric CCTA findings and help prioritize clinical attention. Function-dominant phenotypes may warrant greater emphasis on ischemia-oriented evaluation and closer follow-up, whereas structure-dominant phenotypes may highlight the need for intensified plaque-stabilizing and risk factor-modifying strategies. By contrast, the inflammation-dominant phenotype remains primarily exploratory, given the weak and unstable prognostic signal observed in the present cohort.

Accordingly, the potential clinical value of this framework lies in refining the interpretation of coronary risk heterogeneity rather than replacing guideline-based management. Prospective validation is required before this approach can be incorporated into routine clinical use.

Several limitations should be acknowledged. First, this was a single-center retrospective study, which may limit the generalizability of the findings. Second, the phenotype thresholds were cohort-derived and internally standardized. Although this strategy improved internal interpretability, it may limit reproducibility and external validity. Third, the discretization of continuous variables may have resulted in some loss of information. In addition, because functional, structural, and inflammatory abnormalities frequently coexist, the rule-based mutually exclusive phenotype classification may have oversimplified overlapping pathophysiological processes and introduced some degree of misclassification bias. However, additional sensitivity analyses using a non-mutually exclusive modeling approach yielded consistent results for the functional and structural domains, suggesting that the impact of this simplification on the main conclusions is limited. Fourth, although standardized acquisition protocols and analysis software were used, CT-FFR, plaque quantification, and FAI measurements may vary across scanners, reconstruction algorithms, and post-processing platforms. Fifth, despite parsimonious modeling and penalized regression, residual overfitting cannot be completely excluded given the modest number of events. Finally, the relatively small inflammation-dominant subgroup and limited number of events may have reduced statistical power and contributed to imprecise effect estimates. Larger multicenter prospective studies are therefore required to validate and refine this phenotype framework.

## 5. Conclusions

Distinct imaging-defined pathological axes showed non-equivalent prognostic associations in this cohort, with the function-dominant phenotype showing the strongest observed risk signal, followed by the structure-dominant phenotype. The prognostic significance of isolated inflammation-dominant phenotype remained uncertain after multivariable adjustment because the corresponding estimate was imprecise. These findings support a pathophysiology-oriented interpretation of multiparametric CCTA in NOCAD. However, given the exploratory nature of the classification and the use of internally derived thresholds, prospective external validation is required before broader clinical application.

## Figures and Tables

**Table 1:** Patients’ Baseline Characteristics.

Variables	MACE Group (*n* = 56)	Non-MACE Group (*n* = 429)	*p* Value
Baseline characteristics			
Age, years	68.4 ± 7.8	67.8 ± 7.1	0.502
Sex (male), *n* (%)	26 (46.4)	186 (43.4)	0.680
BMI, kg/m^2^	23.8 ± 3.8	23.7 ± 3.6	0.654
Cardiac risk factors			
Hypertension, *n* (%)	32 (57.1)	221 (51.5)	0.441
Diabetes mellitus, *n* (%)	15 (26.8)	83 (19.3)	0.192
Hyperlipidemia, *n* (%)	22 (39.3)	145 (33.8)	0.409
Smoking, *n* (%)	14 (25.0)	66 (15.4)	0.078
Family history of CAD, *n* (%)	15 (26.8)	103 (24.0)	0.659
Medication			
Aspirin	25 (44.6)	209 (48.7)	0.576
Statin	33 (58.9)	258 (60.1)	0.870
Beta blocker	20 (35.7)	158 (36.8)	0.890
ACEi/ARB	27 (48.2)	201 (46.9)	0.857
Laboratory results			
Creatinine, μmol/L	70.6 ± 18.5	71.4 ± 17.7	0.392
eGFR, mL/min/1.73 m^2^	78.2 ± 21.2	80.9 ± 22.7	0.183
hs-CRP, mg/L	1.5 (0.5, 4.4)	1.3 (0.6, 4.2)	0.108
Total cholesterol, mmol/L	4.8 (3.5, 5.5)	4.6 (3.5, 5.4)	0.379
LDL cholesterol, mmol/L	2.6 (2.1, 3.8)	2.8 (2.1, 3.9)	0.665
HDL cholesterol, mmol/L	1.1 ± 0.3	1.0 ± 0.2	0.743
Triglycerides, mmol/L	1.5 (0.9, 2.2)	1.3 (1.0, 2.1)	0.364

Values are mean ± standard deviation, *n* (%), or median (interquartile range).; ACEi = angiotensin converting-enzyme inhibitor; ARB = angiotensin II receptor blocker; CAD = coronary artery disease; eGFR = estimated glomerular filtration rate; hs-CRP = high-sensitivity C-reactive protein; HDL = high-density lipoprotein; LDL = low-density lipoprotein; MACE = major adverse cardiovascular events.

**Table 2:** Comparison of CCTA Plaque Characteristics, FAI, and CT-FFR.

Variables	MACE Group (*n* = 56)	Non-MACE Group (*n* = 429)	*p* Value
%DS	37.4 ± 7.2	35.1 ± 7.6	0.117
Plaque length (mm)	27.1 ± 7.6	26.2 ± 5.8	0.419
MLA (mm^2^)	5.5 [4.0, 7.2]	6.1 [4.8, 7.8]	0.134
PV (mm^3^)	256.9 [162.8, 348.5]	208.6 [35.9, 304.9]	0.005
Calcified PV (mm^3^)	92.18 [65.8, 134.8]	78.4 [46.1, 127.1]	0.178
Non-calcified PV (mm^3^)	157.6 [113.2, 220.5]	115.7 [75.7, 178.4]	0.004
Total PB (%)	53.1 [46.2, 57.3]	48.2 [42.7, 54.5]	0.005
Calcified PB (%)	18.4 ± 3.6	20.2 ± 3.2	0.314
Non-calcified PB (%)	33.2 [23.1, 38.7]	30.3 [21.5, 34.6]	0.067
FAI (HU)	−76.2 [−81.5, −69.5]	−78.0 [−85.7, −70.4]	0.113
CT-FFR	0.86 [0.80, 0.91]	0.91 [0.85, 0.96]	<0.001
HRP, *n* (%)	11 (19.6)	16 (3.7)	<0.001

Values are mean ± standard deviation, *n* (%), or median (interquartile range). %DS = percentage diameter stenosis; MLA = minimum lumen area; PV = plaque volume; PB = percentage burden; FAI = Fat attenuation index; CT-FFR = fractional flow reserve derived from computed tomography; HRP = high-risk plaque.

## Data Availability

The data that support the findings of this study are available from the corresponding author upon reasonable request due to privacy and institutional restrictions.

## Supplementary Materials

The following supporting information can be downloaded at https://www.mdpi.com/article/10.3390/jcdd13040171/s1, Figure S1. Hazard ratios for functional, structural, and inflammatory domains in the primary and non-mutually exclusive sensitivity models. Table S1. Comparison of primary Cox and estimated Firth penalized Cox regression across sequential models for MACE.

## Abbreviations

The following abbreviations are used in this manuscript:

CCTA	Coronary computed tomography angiography
NOCAD	Nonobstructive coronary artery disease
CT-FFR	CT-derived fractional flow reserve
FAI	Fat attenuation index
PB	Plaque burden
PV	Plaque volume
HRP	High-risk plaque
MACE	Major adverse cardiovascular events
CAD	Coronary artery disease
HR	Hazard ratio
CI	Confidence interval
BMI	Body mass index
HU	Hounsfield unit
