# Supplementary material for: A Pathophysiology-Oriented Imaging Phenotype Framework for Nonobstructive Coronary Artery Disease
Source: J Cardiovasc Dev Dis. 2026 Apr 18;13(4):171. doi: 10.3390/jcdd13040171 (PMC13117013; doi:10.3390/jcdd13040171)
Supplement: Supplementary file 1 [file jcdd-13-00171-s001.zip › jcdd-4206297-supplementary.pdf]

Supplementary Figure S1. Hazard ratios for functional, structural, and inflammatory domains in the primary and non-mutually exclusive sensitivity models.

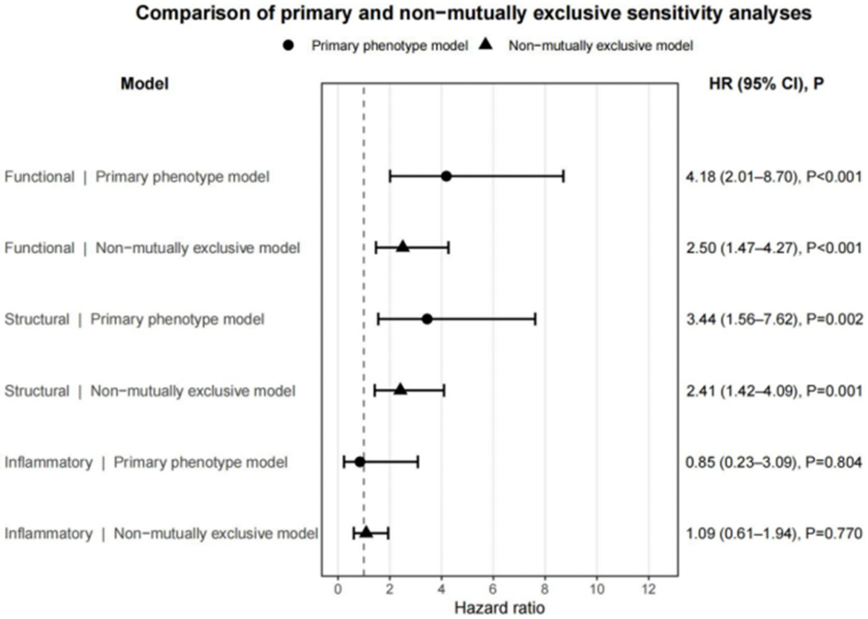

Supplementary Table S1. Comparison of primary Cox and estimated Firth penalized Cox regression across sequential models for MACE.

Model	Comparison	Primary Cox HR (95% CI)	*P* value	Estimated Firth HR (95% CI)*	*P* value*
Model 0 (unadjusted)	Inflammation-dominant	0.87 (0.24-3.16)	0.833	0.88 (0.24-3.18)	0.841
	Structural-dominant	3.58 (1.62-7.89)	0.002	3.36 (1.52-7.41)	0.003
	Functional-dominant	4.58 (2.22-9.48)	<0.001	4.25 (2.06-8.78)	<0.001
Model 1 (+ age and sex)	Inflammation-dominant	0.86 (0.24-3.12)	0.818	0.87 (0.24-3.15)	0.827
	Structural-dominant	3.57 (1.62-7.87)	0.002	3.35 (1.52-7.38)	0.003
	Functional-dominant	4.52 (2.18-9.35)	<0.001	4.19 (2.03-8.67)	<0.001
Model 2 (+ age, sex, hypertension, and diabetes mellitus)	Inflammation-dominant	0.86 (0.23-3.13)	0.816	0.86 (0.24-3.15)	0.824
	Structural-dominant	3.22 (1.45-7.13)	0.004	3.03 (1.37-6.73)	0.006
	Functional-dominant	4.28 (2.04–8.95)	<0.001	3.98 (1.90-8.32)	<0.001
Model 3 (+ statin use)	Inflammation-dominant	0.85 (0.23-3.12)	0.809	0.86 (0.23-3.14)	0.818
	Structural-dominant	3.29 (1.47-7.32)	0.004	3.10 (1.39-6.90)	0.006
	Functional-dominant	4.24 (2.03-8.88)	<0.001	3.95 (1.88-8.26)	<0.001

HR = hazard ratio; CI = confidence interval; MACE = major adverse cardiovascular events. * Reference category: low-risk phenotype.
